# Identifying and addressing gaps in the implementation of a community care team for care of Patients with multiple chronic conditions

**DOI:** 10.1186/s12913-019-4709-6

**Published:** 2019-11-15

**Authors:** Kasey R. Boehmer, Diane E. Holland, Catherine E. Vanderboom

**Affiliations:** 1 Knowledge and Evaluation Research (KER) Unit, Mayo Clinic, 200 First Street SW, Rochester, MN 55905 USA; 2 Nursing Research Division, Department of Nursing, College of Medicine, Mayo Clinic, 200 First Street SW, Rochester, MN 55905 USA

## Abstract

**Background:**

Patients with multiple chronic conditions represent a growing segment for healthcare. The Chronic Care Model (CCM) supports leveraging community programs to support patients and their caregivers overwhelmed by their treatment plans, but this component has lagged behind the adoption of other model elements. Community Care Teams (CCTs) leverage partnerships between healthcare delivery systems and existing community programs to address this deficiency. There remains a gap in moving CCTs from pilot phase to sustainable full-scale programs. Therefore, the purpose of this study was to identify the cognitive and structural needs of clinicians, social workers, and nurse care coordinators to effectively refer appropriate patients to the CCT and the value these stakeholders derived from referring to and receiving feedback from the CCT. We then sought to translate this knowledge into an implementation toolkit to bridge implementation gaps.

**Methods:**

Our research process was guided by the Assess, Innovate, Develop, Engage, and Devolve (AIDED) implementation science framework. During the Assess process we conducted chart reviews, interviews, and observations and in Innovate and Develop phases, we worked with stakeholders to develop an implementation toolkit. The Engage and Devolve phases disseminate the toolkit through social networks of clinical champions and are ongoing.

**Results:**

We completed 14 chart reviews, 11 interviews, and 2 observations. From these, facilitators and barriers to CCT referrals and patient re-integration into primary care were identified. These insights informed the development of a toolkit with seven components to address implementation gaps identified by the researchers and stakeholders.

**Conclusion:**

We identified implementation gaps to sustaining the CCT program, a community-healthcare partnership, and used this information to build an implementation toolkit. We established liaisons with clinical champions to diffuse this information. The AIDED Model, not previously used in high-income countries’ primary care settings, proved adaptable and useful.

## Background

The prevalence of multiple chronic conditions, currently affecting three in four adults 65 and older, is growing [1, 2]. These patients suffer from both a high burden of illness and a high burden of treatment [3]. When illness and treatment burden overwhelm patients’ and their caregivers’ abilities and resources or capacity to self-care, patient outcomes suffer [4]. Recent work has highlighted the need for care processes to emphasize patient-centeredness in treating multiple chronic conditions, and patient-family engagement as a method for making patient-centered care a reality [5]. The Chronic Care Model (CCM) supports the idea of leveraging community programs to support patients and their caregivers in self-managing their chronic conditions [6]. However, this component has lagged behind the adoption of other model elements, such as improving clinical information systems [7, 8]. Additionally, implementations of the CCM have not supported patients’ capacity in some fundamental ways. Elements of capacity support noted missing in CCM interventions include providing practical resources, such as assistance with financial or transportation problems and assisting patients with leveraging their social networks to handle the burden of illness and treatment [9].

Programs that leverage partnerships between healthcare systems and existing community programs address these deficiencies observed in the current structure of chronic care delivery. An early exemplar of such program is Vermont’s “Blueprint for Health,” an initiative that began as a pilot in the state in 2003, which included a community care team at the heart of its healthcare delivery reform [10]. During the Blueprint for Health pilot, the program reduced patient hospitalizations, emergency department visits, and overall costs. It has since been adopted by the majority of primary care practices in Vermont [10, 11]. The state-wide transformation, which occurred in Vermont, was inspirational. However, it has been documented that there are significant challenges to connecting community and healthcare resources. For example, evaluation of community-healthcare system connectivity illustrated that community-based services and healthcare services operate in two separate worlds and perceived that it was not necessarily either parties job to connect or refer to the other [12].

Modeled after Vermont’s Blueprint for Health, the Community Care Team (CCT) was developed and implemented in a county in an upper Midwestern state [13, 14]. The county is served by two healthcare delivery systems, both that have substantial primary care practices. One healthcare system is a large academic medical center and the other is a community hospital medical center. The CCT was developed using three proven approaches to meet the needs of patients with chronic illness: 1) care coordination by either a nurse or social worker, 2) partnerships with existing community services, and 3) the use of the Wraparound process [14, 15].

Briefly, we have summarized the way the program worked during its research evaluation phase and continues to work as it continues to operate in clinical practice. Patients appropriate for the CCT program are adults with chronic health conditions who are overwhelmed. Typically, their struggles are both burden of illness and treatment, and they have been identified as unable to carry out their self-management in full. A care coordinator, social worker, public health nurse, or other primary care clinician can identify eligible patients. After enrollment, a member of the CCT meets patients in their home for a comprehensive assessment of their health and living environment. Then, the CCT holds an initial group meeting with the patient and their support persons at their primary healthcare clinic. During this meeting, the CCT focuses on patient strengths to leverage them to improve self-care and on identifying patient and family priority concerns [16]. Based on the strengths and concerns assessed, the CCT creates with the patient and their support person(s) a shared action plan to address their priority concerns. The action plan includes concrete tasks, delegates each task to a member of the CCT, the patient, or their caregiver, sets up a timeline for completion and follow-up, and indicates the expected results. Additional deliverables of the CCT meeting include a Crisis Prevention Plan and a Circle of Support. The Crisis Prevention Plan indicates patient-identified changes signaling a difficult day and the way they will obtain assistance before the situation spirals out of control. The Circle of Support includes community and informal resources available to assist the patient with self-management activities [13]. A copy of each group meeting proceedings and the Action Plan is made available to all team members, patients, and caregivers at the end of each group session. The work of implementing the Action Plan takes place over the subsequent 12-week period. The CCT meets again with the patient and their caregiver(s) following the 12 weeks to re-evaluate progress toward goals, address new problem areas, and finish any outstanding tasks.

In its pilot evaluation, the CCT showed significant improvement in scores on the Patient Assessment of Chronic Illness Care (PACIC), a standard validated measure of the quality of chronic illness care from the patient’s perspective [13]. Differences in patient-reported health outcomes, such as pain and anxiety/depression were not statistically significant, in part due to the small sample size in the pilot program [13]. However, scores trended toward improvement after the program despite patients reporting higher levels of pain and anxiety/depression at baseline than the control group [13]. Given the strong correlation between the elements of limited physical and emotional capacity and disruption to patients’ lives by their illness and treatment [17], these trends are promising. A recent economic evaluation of the program revealed a decrease in total healthcare costs by 23% for participants [18].

Based on the positive pilot findings and solid stakeholder endorsement of the CCT’s assistance to patients, there was strong interest in moving the CCT from a research-funded pilot program to a sustainable resource available to the local community and primary care practices. However, there were challenges that were identified to making this desire a reality. First, past research indicated challenges in enacting healthcare-community connected partnership [12], and CCT champions acknowledged a lack of awareness about the CCT amongst primary care physicians, care coordinators, and social workers in both healthcare systems. Therefore, there was a need to assess the additional implementation support that was required to support continued referral to the program and to develop the materials and processes needed to aid in that implementation.

## Methods

### Aims

*Therefore, the aims of this study were to identify* (1) *the cognitive and structural needs of clinicians, social workers, and nurse care coordinators to effectively refer appropriate patients to the CCT and* (2) *the value these stakeholders derived from referring to and receiving feedback from the CCT. We then sought to translate these needs and value-propositions into an implementation toolkit to sustain referral to the CCT in the future, particularly as the program was concluding its research phases and transitioning to serving as a clinical service.*

### Ethics review

The Mayo Clinic Institutional Review Board approved all activities. We obtained verbal consent from all participants using an IRB-approved verbal consent script. The Mayo Clinic IRB declared this study minimal risk and therefore, a verbal rather than written consent was acceptable. Participants were given a copy of the verbal consent script for their records and the consent was documented on a secure server in a study log.

### Conceptual model

**Fig. 1:**
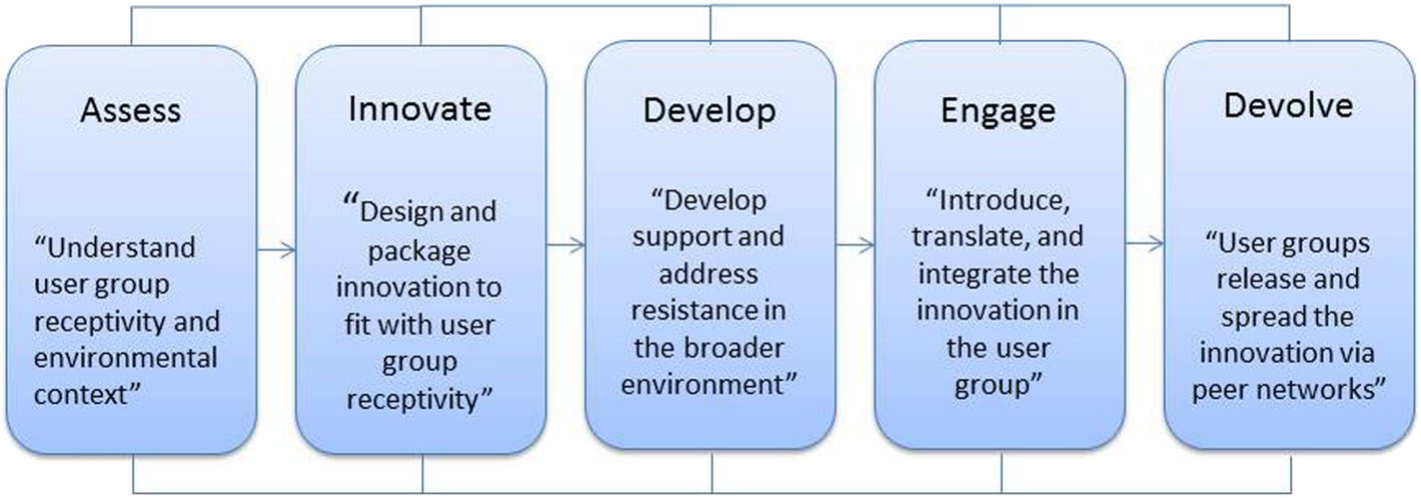
AIDED Model for Implementation and Scale-up

Because our aims were primarily focused on the evaluation of current implementation processes to sustain the CCT in practice, we approached our methods with an implementation science lens [19]. Specifically, we selected an existing implementation science framework, the Assess, Innovate, Develop, Engage, and Devolve (AIDED) Model for Dissemination, Diffusion, and Scale Up (Fig. 1), which is focused on the sustainability and scale-up of existing interventions [20]. This model was developed by the Yale Global Health Leadership Institute, in partnership with the Gates Foundation. Originally, the model was developed for use in low- and middle- income countries to address the lack of wide-scale implementation of interventions proven efficacious on family health outcomes in these countries. These interventions include injectable contraceptives, breastfeeding, and community health worker programs. However, despite the development of AIDED in low- and middle-income countries for family health interventions, it appears more broadly applicable and particularly useful to consider the ways in which innovations can be tailored and spread to meet the needs of other and diverse stakeholders. We, therefore, sought to use the model in a well-resourced healthcare system and community in the United States.

Each step of the AIDED model is conducted in phases but the process is not entirely linear [20]. We used this conceptual model to inform our data collection and analysis process. Additionally, our methods were informed by a user-centered design approach, which focuses on deeply understanding the needs of the user, in this case, healthcare professionals intended to refer patients to the CCT, and iteratively designing products or solutions to their needs [21, 22].

### AIDED process

#### Assess

The AIDED process steps were completed between June 2016 and January 2017. The purpose of the *Assess* phase was to understand what the CCT had accomplished for patients referred to and graduated from the program, as well as to understand the information needs of potential referrers (clinicians, care coordinators, and social workers) to the CCT program. To do a comprehensive assessment we conducted chart reviews of previous CCT patients, conducted an observation of a referral, and an observation of a CCT home visit post-referral. To conduct our chart review, the lead author (KB) was provided a list of patients referred to the program between November 2014 and April 2016 (*n* = 27). Patient clinic identifiers were then scanned through a research ethics database to identify if they were eligible for research chart review; those that were not chart review eligible were excluded (*n* = 1). Patients who completed both program visits (*n* = 16) were then purposefully selected based upon referring individual (typically a care coordinator) and time of referral (early in the program and late). In total, 14 charts were reviewed. CCT visit notes were reviewed and the following information was extracted from the charts using an excel spreadsheet: chronic conditions, precipitating factors to CCT referral, patient capacity needs using a previous classification structure [23], issues of patient treatment burden [24], key takeaways from the first CCT visit and the follow-up CCT visit. KB also took reflexive notes in the excel spreadsheet regarding her takeaway learnings from each chart and from constant comparison of the charts.

Additionally, we conducted 11 interviews with current and potential referral sources to the CCT including three physicians, two CCT staff, four nurse care coordinators, and two social workers. These interviews were used for design purposes, rather than formal qualitative analyses, and therefore, the interviewer (KB) took detailed interview notes and reflections on design implications immediately following the interviews and discussed summary findings every two weeks with co-investigators DH and CV. A sample interview guide is included in Additional file 1. Finally, we conducted two observations, one in the context of a social work visit where a CCT referral might be made and another in the context of a CCT enrollment home visit. These observations were done for design purposes as well, to provide additional contextual information beyond the chart reviews and interviews. KB conducted the observations and took detailed notes while in the field and reflections upon completing the observations.

#### Innovate

We used the information gathered during the *Assess* phase to inform the *Innovate* phase. Specifically, we first summarized our learnings about the referral process, patient successes and struggles, and feedback clinicians were providing. We then met as a team (KB, DH, CV) to discuss these findings, identify key gaps in the overall clinical process of the CCT, from referral to patient graduation and clinician feedback, and proposed potential solutions to these problems. These solutions became the foundation of the implementation toolkit.

During the *Innovate* phase, we worked with stakeholders (referrers to and delivers of the CCT program) to iteratively design the implementation toolkit to support sustainable referrals to and feedback communication from the CCT. Specifically, CV and DH shared toolkit components with CCT stakeholders including referring clinicians, social workers, and care coordinators to elicit feedback. We used feedback to iteratively modify toolkit components.

#### Develop

During the *Develop* phase, we worked to develop stakeholder engagement to prepare potential champions for the CCT referral process for the toolkit’s dissemination. These stakeholders included those consulted during the Assess and Innovate phases and also included clinicians in primary care at-large at the two referring institutions. We continue to work with identified implementation champions to *Engage* them and additional referrers to *Devolve* the toolkit throughout the champions’ social networks.

## Results

### Assess

During the *Assess* phase, our chart review revealed that there was no standardized method to document CCT referrals and CCT program outcomes in the electronic health record. Patient characteristics of chart reviewed patients can be found in Table 1. We also learned that patients were referred for a variety of capacity problems: financial, physical, emotional, and social. All patients had physical capacity problems, but had additional capacity deficits that prompted their referral to the CCT. While the financial and physical capacity issues were certainly complex, the majority of them were fully addressed during the CCT program. Whereas the social and emotional capacity issues were addressed during the CCT, their nature made them more difficult to fully resolve during the program. Because of the varying status of issues addressed for individual patients, the need for clear communication and re-integration into traditional primary care was strongly needed.

**Table 1:** CCT chart review patient characteristics

Age (Median, Range)	65 (38–76)
% Female	50%
Number of conditions (Median, Range)	5.5 (1–9)
Complete listing of conditions for all patients included in chart review	Diabetes, depression, anxiety, meningioma, hypertension, coronary artery disease, hyperlipidemia, migraine, traumatic brain injury, unspecified mood disorder, gastroesophageal reflux disease (GERD), seizures, chronic myeloid leukemia, hypertension, diabetes, atrial fibrillation, hepatitis C, lung disease, arthritis, fibromyalgia, ataxia, COPD, bladder disease, lung cancer, congestive heart failure, obesity, asthma, chronic rhinosinusitis, multiple sclerosis, chronic kidney disease, end-stage renal disease, chronic anemia, liver disease, chronic fatigue, chronic pain

During our interviews, we learned that the lack of identifiable and robust documentation created a barrier to implementation of the program; people who referred to the CCT found it difficult to find and to understand what happened during the CCT process, the status of the patient’s situation at the completion of the program, and what further actions were required on the part of the primary care team. Additionally, clinicians indicated that a strong feedback loop to describe the patients’ successes was also the best promoter of future referrals, based on their past experience with other programs.

Furthermore, through chart review and individual interviews, we also identified three strengths of the program that past referrers felt were not found in any other program offered through the healthcare institution or in the community. These strengths were: 1) the wrap-around nature of the program that supported the patient by engaging caregivers and multiple disciplines in the same meeting; 2) the focus on building upon the patient’s strengths; and 3) the rich compilation of resources that patients could be referred to from the CCT due to pooling the community knowledge into one team.

In contrast to current referrers, interviews with potential CCT referrers highlighted their lack of knowledge about these distinguishing CCT factors. Interviews with potential referrers also emphasized that in-part due to their newness to the program and in-part due to the busy nature of clinical practice for complex patients, they felt uncertain of inclusion criteria for a CCT referral. Furthermore, if their patients met the criteria, they felt uncomfortable with their ability to introduce the program to the patient in a succinct manner.

### Innovate

**Fig. 2:**
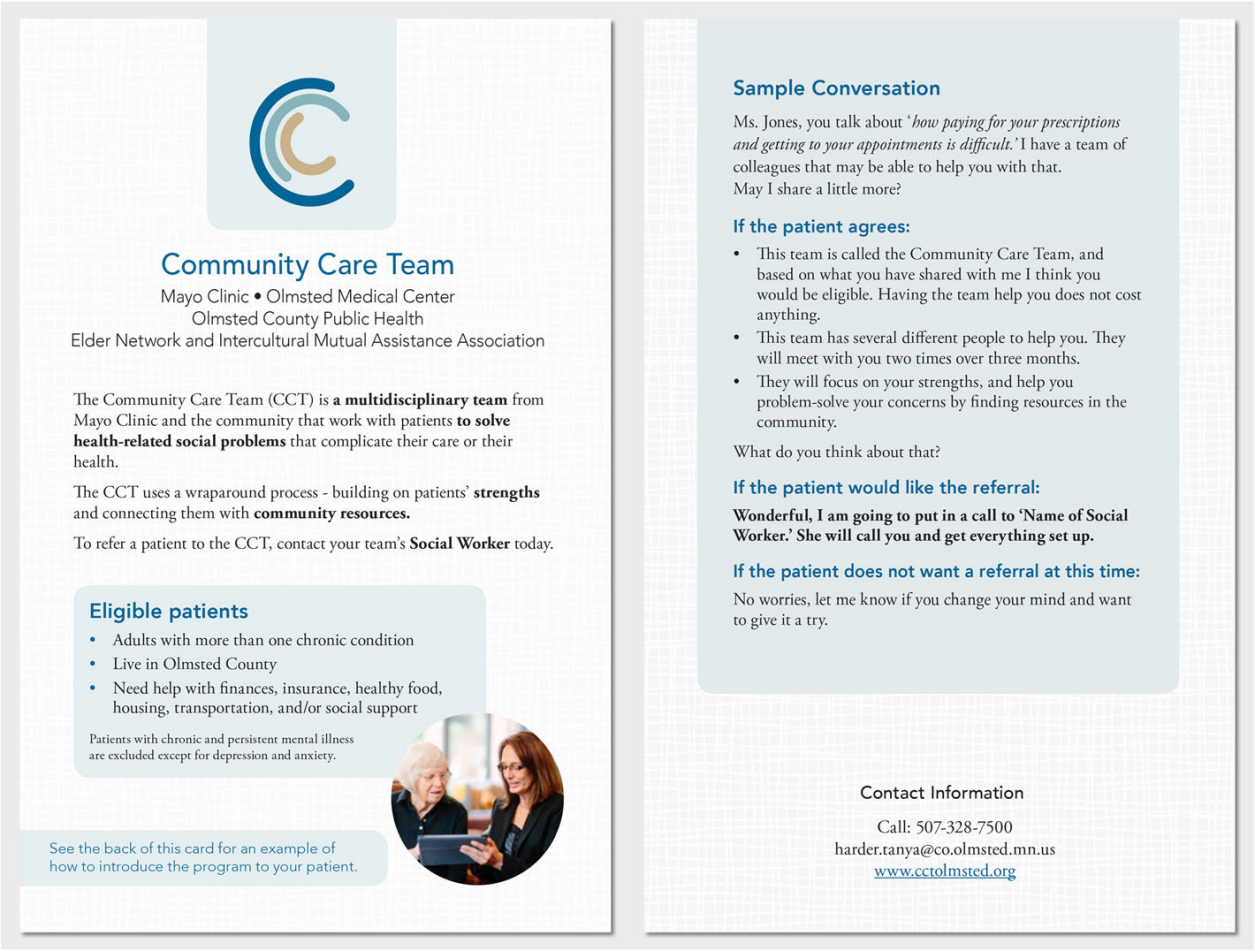
Health Professional Information Postcard

Building on these findings, during the *Innovate* phase, we first worked as a small team to develop an implementation toolkit with seven components to meet the needs of past and future users and fill knowledge gaps that existed. These seven components, which will be described in more detail were: a program executive summary, a clinician information postcard, a five-minute presentation about the program that could be used by anyone promoting the program to local departments, a brief video, a referral process diagram, a patient success stories handout, and a CCT visit standardized documentation template for the electronic health record. We created a referral postcard for health professional use (Fig. 2), which included a description of the purpose of the program, the eligibility criteria, a script for introducing the program to patients, and a contact to make the referral. This toolkit item solved the problem that new referring clinicians were having regarding clear eligibility criteria and comfort in talking to the patients about the program.

Additionally, we summarized the process we used to develop the toolkit, the key findings, and instructions on how to make a referral into a one-page executive summary. For larger group meetings, we summarized information about the CCT and the referral process into a five-minute presentation and created a promotional video about the CCT that was appropriate to introduce patients and health professionals to the program. The presentation included a referral process diagram starting from identification of an appropriate patient for the CCT through the patient being accepted into or denied access to the program. Patients are rarely denied access to the program, but if such instance occurred, a reason why the patient was not accepted would be provided to the referrer. Ultimately, this process diagram was used to improve the feedback process from the CCT team members to the referring health professional and the patient’s primary care team. This diagram could also be used independently to ensure referring health professionals understood the process. To promote referrals, we created a one-page document highlighting the story of two CCT patients (Additional file 2), including the reason for their referral to the CCT and the positive outcomes from their participation in the program. Finally, our toolkit included a standard template to document CCT visits in the patient record that was easy to find and to understand what happened during the CCT process, ensuring the feedback loop from referral to conclusion of the final CCT visit, and solving the initial problem identified in our chart reviews of unclear documentation.

### Develop

During the *Develop* phase, DH and CV met individually and in groups with clinical stakeholders that were likely to use the toolkit to sustain the CCTs referrals into the future. These included those consulted during the Assess and Innovate phases plus other clinicians likely to refer to the CCT in primary care at referring institutions. Feedback from these stakeholders was solicited regarding all toolkit elements and this was communicated back to KB to modify toolkit components based on stakeholders’ needs. As mentioned previously in the methods section, the *Engage* and *Devolve* steps remain ongoing.

## Discussion

We used the AIDED Model to examine the implementation gaps, user-needs, and stakeholder-perceived value of the CCT. Ultimately, this led us to the development of an implementation toolkit with seven unique components to create support for sustainable referrals to the CCT from primary care clinicians. Each toolkit component met an implementation gap identified through our AIDED process. For example, the documentation template for CCT visits was designed to address the lack of uniformity in reporting uncovered through the chart review and echoed by referring clinicians who expressed confusion about outcomes from the program in their interviews. This process also served to strengthen the feedback loop from the CCT to the primary care team after patients complete the 12-week program.

Before considering the strengths of this work, we must acknowledge its limitations. First, the included work was conducted at the completion of pilot funding for the program to support its transition to a clinical resource. Because of this, additional patient outcomes are not available beyond what was reported in the earlier pilot publication [13]. Second, the most critical limitation is that this work conducted to scale up the CCT occurred in one of the two healthcare institutions in the upper Midwest the CCT served. This institution was a well-resourced academic medical center. Therefore, the findings may not be applicable in other settings with different geographic characteristics or in which implementation is spread across multiple healthcare institutions within a larger region. However, we expect that given the flexibility of the AIDED model, others would be able to use it to tailor the toolkit materials to fit their own similar programs in different settings. In spite of this limitation, successful use of the AIDED model and expansion of the CCT are two important contributions to implementation science and to healthcare delivery for patients with chronic conditions.

The AIDED model was originally developed for use to scale up health interventions in low- and middle-income countries with limited implementation resources [20]. To date, it has been applied only to those settings [25, 26], although one study protocol describes using it in the U.S. healthcare system [27]. However, its tenets – assessing context, innovating intervention components and delivery within the current context, developing support and addressing resistance within the broader environment, engaging the key user groups, and devolving through the social networks of users [20] – were informative in a well-resourced healthcare system and community. These findings suggest that AIDED may be broadly applicable to scale-up interventions and to address barriers to implementation in diverse settings. Specifically, in translational science, we often seek to implement programs and interventions exactly as they were developed and proven successful in pilot studies. AIDED suggests that researchers must be more flexible and nimble in addressing the *context* of the larger environment [20]. “Successful scale-up is not fully under the control of the innovator, donor, or implementer but rather grows organically out of a deep understanding of and engagement with user groups and their environmental contexts.” [20] Future research should seek to implement AIDED in a variety of contexts internationally to see if its tenets continue to inform successful scale-up projects, regardless of setting.

Second, the CCT program’s strength is that it integrates healthcare and community resources to address needs that are not often met by other contemporary chronic care programs [7–9]. Specifically, it leverages community-healthcare partnership and improves patient capacity by providing tangible resources, such as assistance with finding transportation and investigating financial matters also not observed in CCM implementation. These CCT practices move chronic care towards a Minimally Disruptive Medicine model of care that seeks to pursue patient’s goals with the least possible healthcare footprint on their lives [3, 28]. Importantly, this support for patient capacity may impact patients’ health outcomes. For example, interventions to reduce 30-day readmissions that provided rich support for patients’ capacity were more effective than interventions that provided little or no support for patients’ capacity [29]. Future research should seek to prospectively test the full-scale implementation of the CCT and of other similar programs to understand their impact on other patient-important outcomes, such as health status, readmissions, and overall quality of life.

## Conclusion

In conclusion, we successfully created an implementation toolkit for the Community Care Team (CCT) program that will serve to improve referrals to the program and strengthen the feedback primary care teams receive from the CCT. The application of the AIDED model for intervention scale-up proved helpful in a well-resourced healthcare system, and the expanded CCT referral base should serve to improve the lives of patients with chronic conditions. Future research should seek to continue to test the AIDED model’s success in novel contexts, and to test if a full-scale CCT program results in improved patient health and healthcare systems outcomes.

## Supplementary information

**Additional file 1.** Interview Guides.

**Additional file 2.** Patient Success Stories Handout.

## Data Availability

Interview and observation notes are available by contacting the corresponding author.

## Abbreviations

AIDED: Assess, innovate, develop, engage, devolve
CCM: Chronic care model
CCT: Community care team
