# Supplementary material for: Identifying and addressing gaps in the implementation of a community care team for care of Patients with multiple chronic conditions
Source: BMC Health Serv Res. 2019 Nov 15;19:843. doi: 10.1186/s12913-019-4709-6 (PMC6858771; doi:10.1186/s12913-019-4709-6)
Supplement: Supplementary file 1 — Additional file 1. Interview Guides. [file 12913_2019_4709_MOESM1_ESM.docx]

**Chart Specific**

1. In reviewing this patient’s chart, we saw that they had ongoing Social Work and Care Coordination contacts. Can you tell me a little bit about what informed the referral to the CCT?

- Is there anything that stands out to you about this referral?
- What were you hoping will be improved by the referral?
- Was there a specific need you thought could be better addressed by referral than in the visit with you?

**General**

1. Tell me what you know about the Community Care Team Service.

- What information is key in helping you consider a referral?
- How did you come to know about the CCT?
- How is the CCT helpful?
- In what ways is the CCT not helpful?
- How do you assess if you want to continue referring patients to the CCT?

1. Tell me about the services to which you currently refer patients with chronic conditions and known problems with health-related social needs.

- How did you come to know about these services?
- How are these services helpful?
- In what ways are these services frustrating?
- How do you assess if you want to continue referring patients to these services?
- How are the services you mention different from each other/CCT?

1. How do you come to know that a patient has problems with health-related social needs?

- What does that process feel like?
- What do you then do with that information?
