# Supplementary material for: Identifying and addressing gaps in the implementation of a community care team for care of Patients with multiple chronic conditions
Source: BMC Health Serv Res. 2019 Nov 15;19:843. doi: 10.1186/s12913-019-4709-6 (PMC6858771; doi:10.1186/s12913-019-4709-6)
Supplement: Supplementary file 2 — Additional file 2. Patient Success Stories Handout. [file 12913_2019_4709_MOESM2_ESM.docx]

**Mr. W has Leukemia, type 2 Diabetes - uncontrolled, back pain and limited health literacy. He had the following limitations to his capacity to care for himself:**

- *Financial* - limited resources and problems with Rx Drug Coverage
- *Environmental* - Unable to cook in own home due to broken refrigerator and stove. Relied on coupons for fast food or local restaurants to get 2 for 1 deals.
- *Physical* - When his back pain was bad, he would stay in bed. His diabetes was uncontrolled.

**The CCT did the following to address Mr. W’s needs:**

- *Financial* – Offered assistance with paperwork to help get the assistance he needed for insurance coverage. Attended a Medicare meeting with him and found out he had coverage but was behind in payments thus needed to pay the back amount to avoid a penalty. He was able to start on his insulin medications at out of pocket cost of $25 and get a diabetes testing machine, strips and insulin all under $50.
- *Physical* - He met with a pharmacist and dietician as well as diabetic educator to receive education on insulin management, hypoglycemia and treatment.
- *Environmental* – By getting his medical finances in order, he could afford to fix his kitchen appliances.

**The overall outcome for Mr. W was:**

- At the 2nd CCT meeting, Mr. W looked like a new man. His hair was freshly cut. He was taking insulin twice daily with his meals. His refrigerator had been fixed and he was grilling out and eating more “clean food.” He was more active. We continue to adjust insulin and work on improving diet.

**Ms. L is a patient with multiple chronic conditions including CHF, Diabetes, and COPD. She had the following limitations to her capacity to care for herself:**

- *Social* – Ms. L was living with family in a shared home that was a source of stress and limited her ability to care for herself. She is socially isolated from positive influences in her life.
- *Environmental* – Ms. L was at risk for falls in her current home.

**The CCT did the following to address Ms, L’s needs:**

- *Social* – The CCT helped Ms. L find an apartment that was in her budget. The CCT helped her get an RX for a therapy dog, so that she could keep her dog with her at her new apartment. The CCT connected her with a companion through the Elder Network.
- *Environmental* – The CCT helped Ms. L obtain bedrails to ensure her safety in her new apartment when she sleeps.

**Overall, the outcome for Ms. L was:**

- A much improved living situation from where she was previously, which provides a better environment for her to care for herself.
